# Acute zonal occult outer retinopathy: optical coherence tomography angiography findings and treatment response

**DOI:** 10.3205/oc000203

**Published:** 2022-06-09

**Authors:** Erhan Özyol, Pelin Özyol

**Affiliations:** 1 SANKO University, Faculty of Medicine, Department of Ophthalmology, Gaziantep, Turkey

**Keywords:** acute zonal occult outer retinopathy, optical coherence tomography, optical coherence tomography angiography

## Abstract

Acute zonal occult outer retinopathy (AZOOR) is a rare condition primarily affecting the outer retina based on electrophysiologic studies. In addition to characteristic fundus appearance, spectral domain optical coherence tomography and fundus autofluorescence are unique in diagnosing AZOOR. There are a few reports on optical coherence tomography anjiography (OCTA) in AZOOR disease. In this report, we present a case using multimodal imaging including OCTA and treatment response to combined systemic antiviral, antiinflammatory, and immunosuppressive drugs. En-face OCTA outer retinal slab may provide useful tips for diagnosis and treatment response.

## Introduction

Acute zonal occult outer retinopathy (AZOOR), first reported by Gass in 1992 [1], typically affects young adult women who present with central vision loss with or without photopsia due to rapid loss of one or more large zones of outer retinal function. The exact pathogenesis of the disease is not well known. Infectious, autoimmune, or inflammatory etiologies were supposed to be responsible in the emergence of disease [1], [2]. Few studies have described the role of multimodal imaging in AZOOR [3], [4]. A common consensus has not been achieved for treatment in the literature.

We have evaluated clinical features of AZOOR in a 64-year-old man using multimodal imaging including optical coherence tomography angiography (OCTA) (AngioVue, RTVue XR Avanti; Optovue, Inc., Freemont, CA, USA) and treatment response to combined systemic antiviral and immunsupressive drugs during follow-up.

## Case description

**Figure 1:**
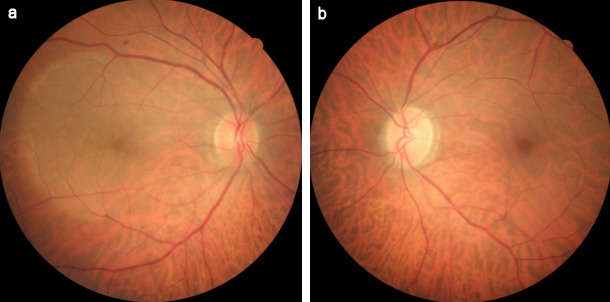
Fundus images at the time of diagnosis (a) Fundus appearance of the AZOOR lesion as an annular ring of yellowish reflex with an about 4–5 disc diameter in the right eye, (b) fundus photo showing the normal left eye

**Figure 2:**
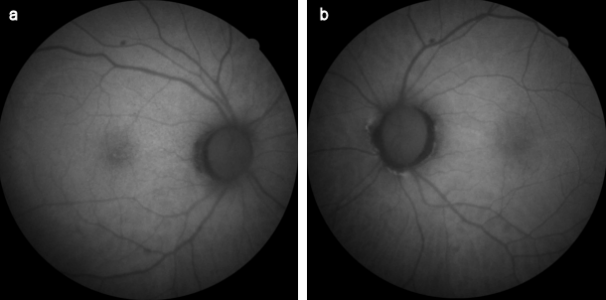
FAF images at the time of diagnosis (a) Slightly increased autofluorescence, more prominent at the border on FAF imaging in the right eye, (b) normal autofluorescence image of the left eye

**Figure 3:**
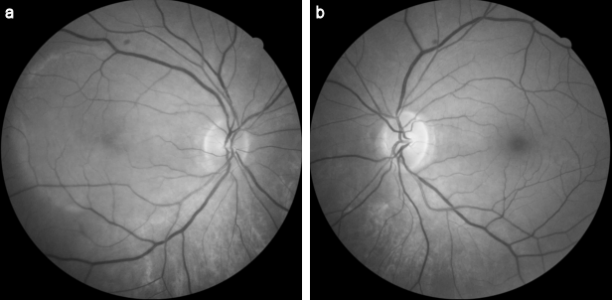
Infrared (IR) images at the time of diagnosis (a) IR image of the right eye showed the lesion contour consistent with the FAF and color images, (b) IR image of the left eye

**Figure 4:**
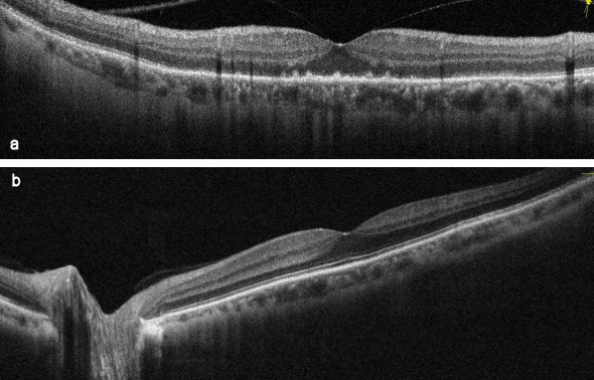
SD-OCT images at the time of diagnosis (a) Spectral domain optical coherence tomography (SD-OCT) revealed hyperreflective dots between RPE and the ellipsoid zone (EZ) and loss or irregularity of the EZ, (b) SD-OCT image of the left eye

**Figure 5:**
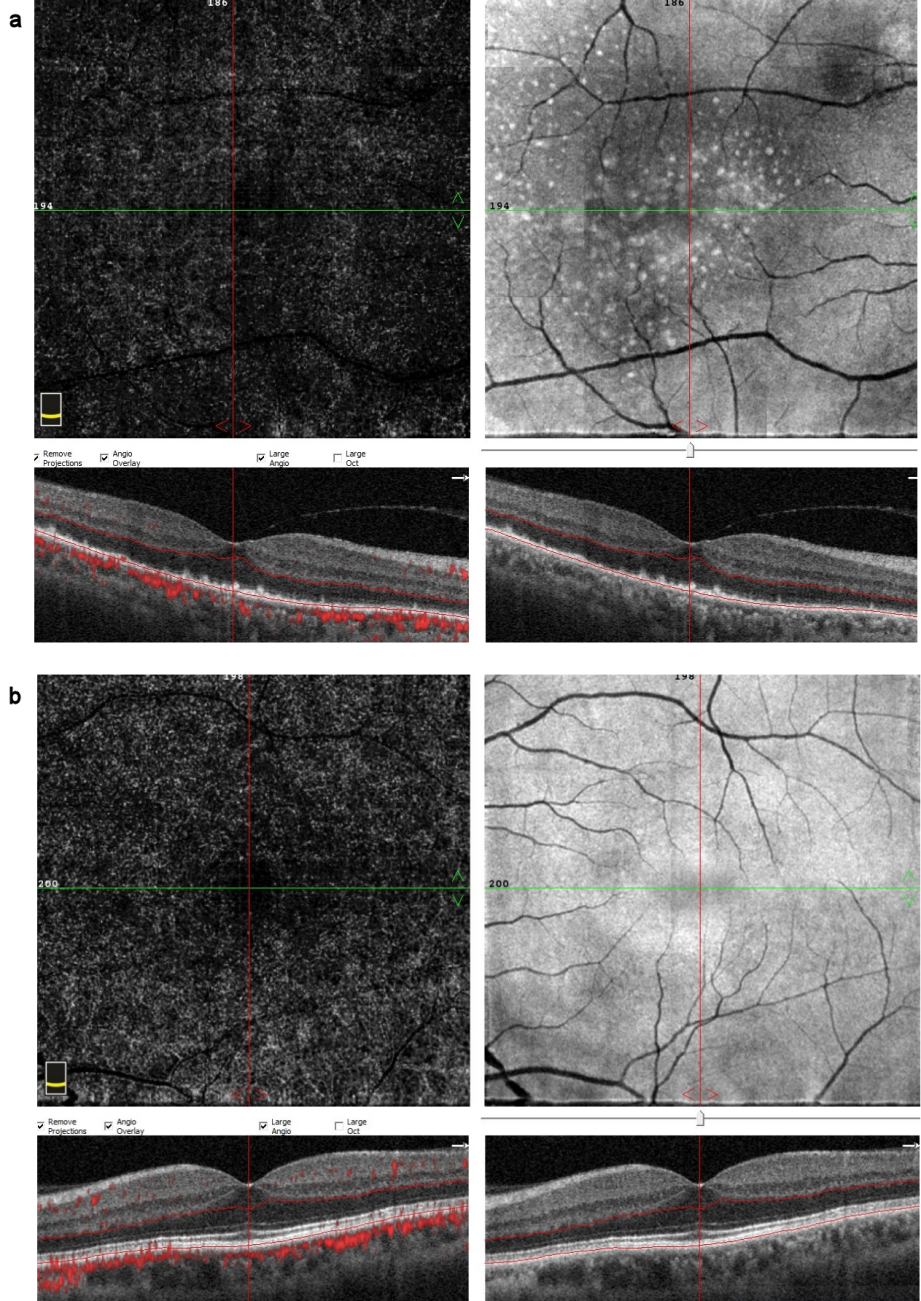
OCTA images at the time of diagnosis (a) Multiple hyperreflective dots in a starry-sky appearance on the en-face OCTA outer retinal slab of the right eye, (b) OCTA image of the left eye

**Figure 6:**
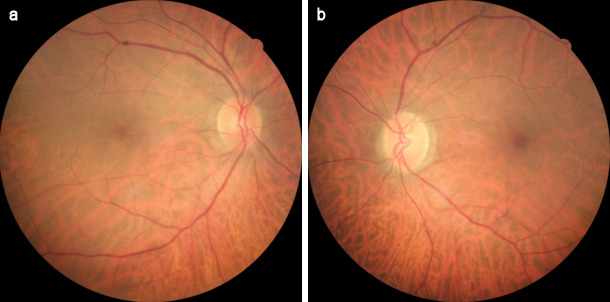
Fundus images at 2-week examination after the treatment (a) The retinal lesion with the annular ring of yellowish reflex disappeared on the color fundus image of the right eye, (b) view of left fundus

**Figure 7:**
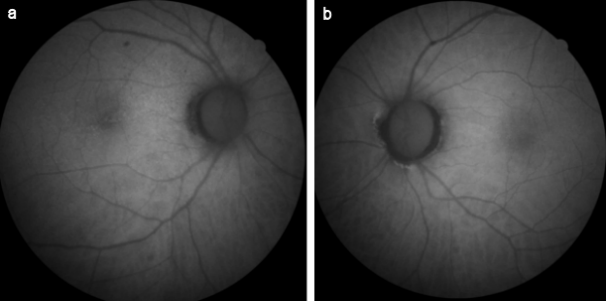
FAF images at 2-week examination after the treatment (a) FAF image of the right eye, (b) FAF image of the left eye

**Figure 8:**
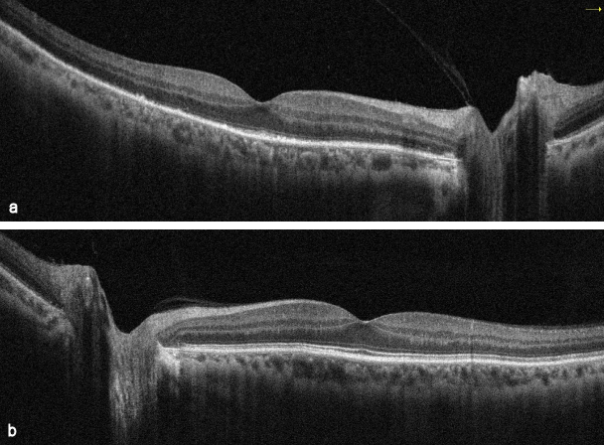
SD-OCT images at 2-week examination after the treatment (a) Marked recovery in EZ and outer segment of photoreceptors on SD-OCT images in the right eye, (b) SD-OCT image of the left eye

**Figure 9:**
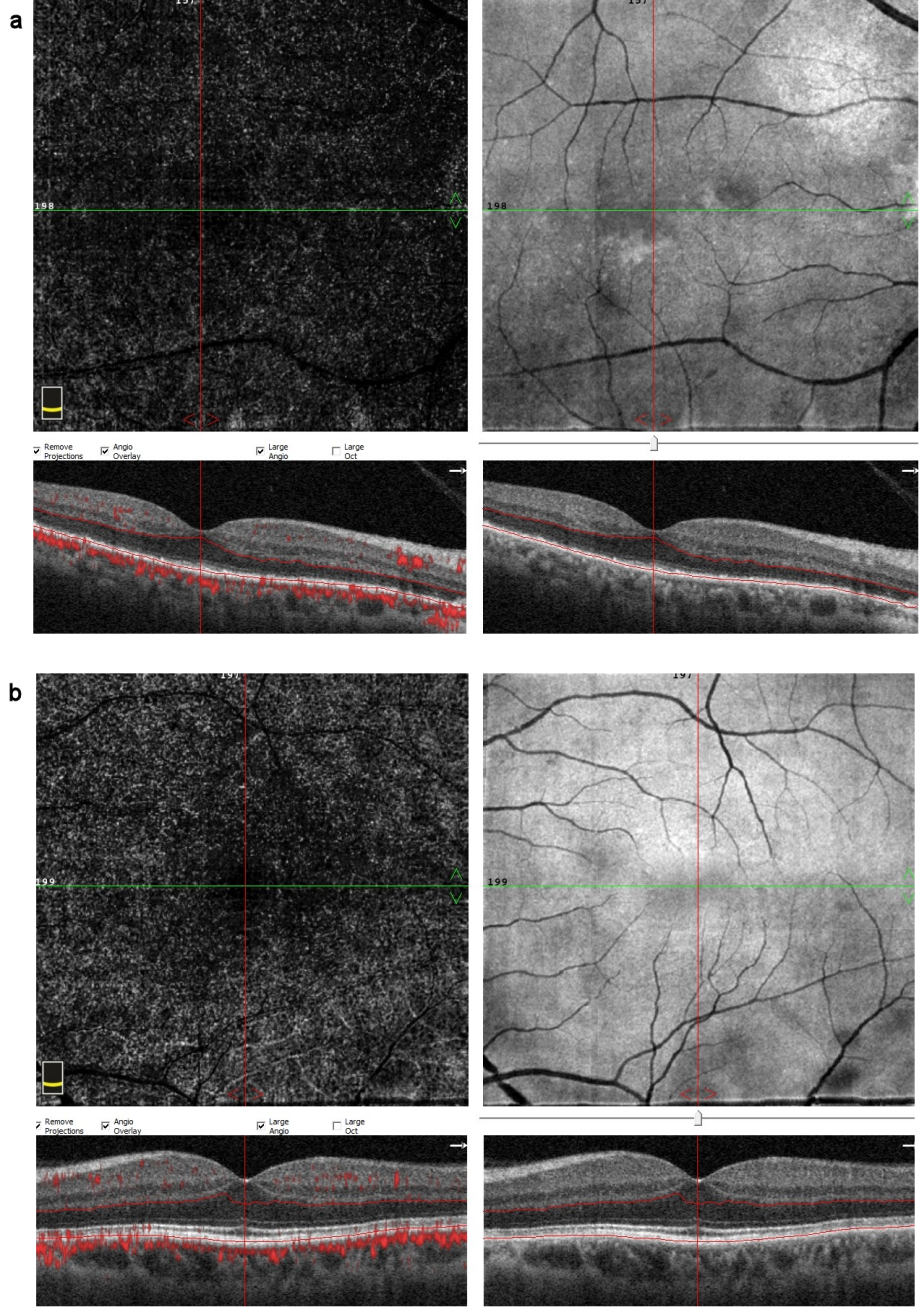
OCTA images at 2-week examination after the treatment (a) An obvious decrease in the hyperreflective dots on en-face OCTA, (b) en-face OCTA image of the left eye

A 64-year-old man presented with sudden onset decrease in vision and photopsia in the right eye since 10 days. Systemic and ocular history was normal. There was no viral infection history preceding the symptoms. The best corrected visual acuity (BCVA) in the patient’s right eye was counting fingers at 2 m and 9/10 in his left eye. There was nuclear sclerosis in both eyes. Fundus examination demonstrated an annular ring of yellowish reflex with an about 4–5 disc diameter at the posterior pole in the right eye (Figure 1 (Fig. 1)). Anterior uveitis or vitritis was not detected. There was slightly increased hyperautofluorescence, more prominent at the temporal border on fundus autofluorescence (FAF) imaging (Figure 2 (Fig. 2)). Infrared fundus image showed the lesion contour consistent with the FAF and color images (Figure 3 (Fig. 3)). Spectral domain optical coherence tomography (SD-OCT) revealed hyperreflective dots between the retinal pigment epithelium (RPE) and the ellipsoid zone (EZ) and loss or irregularity of the EZ (Figure 4 (Fig. 4)). En-face OCTA outer retinal slab demonstrated multiple hyperreflective dots in a starry-sky appearance (Figure 5 (Fig. 5)). The patient was diagnosed as AZOOR and started on oral valgancyclovir (1 g three times daily for a week, once a day for 3 weeks), oral azathioprine (50 mg three times daily) and a weekly tapering course of oral prednisolone (starting at 1 mg/kg body weight). At 2-week examination, significant visual and anatomical improvements were observed. BCVA in the right eye improved to 6/10. The retinal lesion with annular ring of yellowish reflex disappeared (Figure 6 (Fig. 6), Figure 7 (Fig. 7)). There was a marked recovery in the EZ and the outer segment of the photoreceptors on SD-OCT images (Figure 8 (Fig. 8)). En-face structural OCTA images showed an obvious decrease in the hyperreflective dots (Figure 9 (Fig. 9)). The patient was kept under the same treatment and the follow-up continued. The left eye was not affected during the follow-up.

## Discussion

Although AZOOR was first described in 1992 [1], its etiopathogenesis and treatment remain unclear. Based on the electrophysiologic studies, the primary lesions in AZOOR were suggested as the dysfunction and/or degeneration of the photoreceptor outer segment [5].

The fundus view may be normal or damaged at different levels, depending on the stage of the disease and the location of the lesion [6], [7], [8]. However, some features were reported as characteristic, including a demarcating line of the progression at the level of the outer retina and a trizonal pattern of sequential involvement of the outer retina, retinal pigment epithelium, and choroid, as well as frequent zonal progression. AZOOR can manifest as unilateral and asymmetric lesions that have a delineating line to segregate the normal fundus from the AZOOR lesion [4].

Supporting the characteristic color fundus images with SD-OCT and FAF images is very important in the diagnosis of AZOOR. OCTA is a non-invasive, innovative technology for imaging the microvasculature of the retina and the choroid. OCTA has been reported to be useful in the diagnosis and understanding of many retinal conditions [9].

There are a few reports on OCTA in AZOOR disease in the literature. In the first report by Levison et al. in 2016 [10], OCTA was used only to confirm the presence of choroid neovascular membrane in AZOOR. In another case reported by Naik et al. in 2018 [11], the structural en-face OCTA images demonstrated the hyper-reflective dot structures leading to a starry-sky view at presentation. The authors postulated these hyper-reflective dots as degenerating photoreceptor segments. Mehrotra et al. [12] presented an AZOOR case with an orange-yellow-colored demarcation line in the peripapillary area. In that case, the OCTA panorama showed an increased decorrelation signal at the deeper capillary plexus along with the projection artifacts of the superficial capillary plexus. The authors have postulated that an OCTA panorama centered on the fovea with a wider field of view can prove as a potential imaging marker and can subserve as an additional tool to FAF to accurately diagnose and manage AZOOR. In our case, the OCTA findings were in agreement with the report by Naik et al. [11]. Hyperreflective dots in a starry-sky appearance on en-face OCTA outer retinal slab may be characteristic when evaluated with other typical findings both in diagnosis and treatment response.

There are some treatment approaches including observation without any treatment, immunosuppressive agents, systemic or intravitreal steroids, and antiviral therapy, and the results were reported in contradiction to one another [6], [13], [14], [15], [16], [17], [18]. In a study of 51 patients reported by Gass et al. [13], systemic corticosteroids were used in 39 of 113 instances of acute visual loss. The authors reported no difference between treated and non-treated patients. In the same study, systemic steroid and acyclovir or valacyclovir were prescribed for 11 episodes without apparent therapeutic benefit. The authors concluded that there was no treatment of proven value. In a case report by Hoang et al. [14], treatment with antiviral and immunomodulatory drugs failed to control the disease progression. Rodriguez-Coleman et al. [6] reported a case that was given a treatment trial of systemic steroids and acyclovir with no clinical benefit. In contrast, Kitakawa et al. [15] reported a case treated with pulse steroids, followed by oral prednisolone tapered over one month, and improvements in visual acuity and imaging findings after fourteen months. Chen et al. [16] indicated beneficial results of systemic steroids in a retrospective study with a mean follow-up of 47 months. In a retrospective case series, disease stability or improvement was reported after intravitreal steroids. It was noted that treatment-related ocular side effects such as cataract, ocular hypertension, and central serous retinopathy were remarkable [17]. Mahajan and Stone [18] reported 3 cases with a presumptive diagnosis of AZOOR that were responsive to oral valacyclovir without steroid use. Nakao et al. [19] presented a case diagnosed with AZOOR with spontaneous remission without any treatment after two months.

The predictability of the disease is low due to insufficient data. Referring to recent publications, stabilization has been reported to have occurred within 6 months in the majority of patients [13], [20]. However, in a small percentage of cases, the disease continued to worsen after 6 months [21]. In our case, significant improvement in both anatomic and visual function was detected in the second week after combined drug treatment. This improvement continued up to the 2-month examination.

The limitation of the case report is the lack of the anterior chamber or vitreous tap for detecting viral etiology. Although the cause of the disease is unknown, autoimmunity or viral causes are considered in the foreground [13], [22]. Gass et al. [13] suggested several reasons for viral etiology, including the asymmetrical nature of the disease; the lack of response to the administration of corticosteroids; the lack of correlation between a history of autoimmune conditions, bilateral disease and disease severity; the absence of a family history of AZOOR; and the absence of circulating retinal antibodies in many patients. We also know that many autoimmune disorders tend to affect young women [23]. In our case, we thought about a possible viral etiology because of the patient’s age and gender, the absence of a systemic history of autoimmune diseases, the asymmetric nature of retinal involvement, and the unilateral presentation of the disease. Because of this, valgancyclovir was added to the prescription. Despite the potential to be bilateral, the second eye was not involved during the follow-up period. The choroid was not affected during follow-up. Both unilateral involvement and limited zonal damage may be associated with early diagnosis and initiation of broad-spectrum systemic therapy. Since immunosuppressive agents take many weeks to have an effect, improvement of the clinical findings in the early weeks are most probably due to the systemic steroids. Valgancyclovir may contribute to the absence of a late worsening of the clinical findings at two months. However, this needs to be verified. Surely, a spontaneous remission is also possible. Even if the treatment described has led to improved findings, this does not mean that there is a causal relationship between diagnosis and therapy.

Undoubtedly, further studies with a longer follow-up and a larger series are needed to reach a conclusion on etiology and treatment. In addition to other diagnostic tools, OCTA can provide lock tips in diagnosis and follow-up in AZOOR.

## Notes

### Competing interests

The authors declare that they have no competing interests.
